# Inhaled drugs to reduce exacerbations in patients with chronic obstructive pulmonary disease: a network meta-analysis

**DOI:** 10.1186/1741-7015-7-2

**Published:** 2009-01-14

**Authors:** Milo A Puhan, Lucas M Bachmann, Jos Kleijnen, Gerben ter Riet, Alphons G Kessels

**Affiliations:** 1 Horten Centre for Patient Oriented Research and Knowledge Transfer, University of Zurich, Switzerland; 2 Department of Epidemiology, Johns Hopkins Bloomberg School of Public Health, Baltimore, Maryland, USA; 3 Kleijnen Systematic Reviews Ltd, York, UK; 4 Academic Medical Center, Dept. General Practice, University of Amsterdam, 1105 AZ Amsterdam, the Netherlands; 5 Medical Technology Assessment and Epidemiology, Clinical Epidemiology, Clinical Pharmacy, University Hospital Maastricht, Maastricht, the Netherlands

## Abstract

**Background:**

Most patients with chronic obstructive pulmonary disease (COPD) receive inhaled long-acting bronchodilators and inhaled corticosteroids. Conventional meta-analyses established that these drugs reduce COPD exacerbations when separately compared with placebo. However, there are relatively few head-to-head comparisons and conventional meta-analyses focus on single comparisons rather than on a simultaneous analysis of competing drug regimens that would allow rank ordering of their effectiveness. Therefore we assessed, using a network meta-analytic technique, the relative effectiveness of the common inhaled drug regimes used to reduce exacerbations in patients with COPD.

**Methods:**

We conducted a systematic review and searched existing systematic reviews and electronic databases for randomized trials of ≥ 4 weeks' duration that assessed the effectiveness of inhaled drug regimes on exacerbations in patients with stable COPD. We extracted participants and intervention characteristics from included trials and assessed their methodological quality. For each treatment group we registered the proportion of patients with ≥ 1 exacerbation during follow-up. We used treatment-arm based logistic regression analysis to estimate the absolute and relative effects of inhaled drug treatments while preserving randomization within trials.

**Results:**

We identified 35 trials enrolling 26,786 patients with COPD of whom 27% had ≥ 1 exacerbation. All regimes reduced exacerbations statistically significantly compared with placebo (odds ratios ranging from 0.71 (95% confidence interval [CI] 0.64 to 0.80) for long-acting anticholinergics to 0.78 (95% CI 0.70 to 0.86) for inhaled corticosteroids). Compared with long-acting bronchodilators alone, combined treatment was not more effective (comparison with long-acting beta-agonists: odds ratio 0.93 [95% CI 0.84 to 1.04] and comparison with long-acting anticholinergics: odds ratio 1.02 [95% CI 0.90 to 1.16], respectively). If FEV_1 _was ≤ 40% predicted, long-acting anticholinergics, inhaled corticosteroids, and combination treatment reduced exacerbations significantly compared with long-acting beta-agonists alone, but not if FEV_1 _was > 40% predicted. This effect modification was significant for inhaled corticosteroids (*P *= 0.02 for interaction) and combination treatment (*P *= 0.01) but not for long-acting anticholinergics (*P *= 0.46). A limitation of this analysis is its exclusive focus on exacerbations and lack of FEV_1 _data for individual patients.

**Conclusion:**

We found no evidence that one single inhaled drug regimen is more effective than another in reducing exacerbations. Inhaled corticosteroids when added to long-acting beta-agonists reduce exacerbations only in patients with COPD with FEV_1 _≤ 40%.

## Background

Chronic obstructive pulmonary disease (COPD) has become a leading cause of death worldwide and its management requires an enormous amount of human and financial resources [1,2]. Reduction of exacerbation rates is one of the main treatment goals in COPD management since exacerbations bear heavily on the patient's health-related quality of life and prognosis as well as on COPD-related costs [3]. Several conventional meta-analyses provided evidence that long-acting beta-agonists, long-acting anticholinergics and inhaled corticosteroids reduce exacerbations in patients with COPD when compared with placebo [4-6]. The conventional meta-analyses are, however, less informative about the comparative effectiveness of long-acting beta-agonists and anticholinergics or about the additional value of inhaled corticosteroids when added to long-acting bronchodilators. The comparative effectiveness is of great interest to physicians because the predominant question in clinical practice is to choose between treatments rather than deciding whether to treat or not to treat [7,8].

Opinions differ about whether a long-acting bronchodilator alone is sufficient or if an inhaled corticosteroid provides additional benefits at least for some patients [3,9,10]. Any added benefit of corticosteroids, if present, should outweigh the associated risk for adverse effects and their additional costs. Conventional meta-analyses [4-6,11,12] do not provide enough support to solve this debate because evidence from randomized head-to-head comparisons is often unavailable. Also, conventional meta-analyses cannot provide effect estimates for comparisons of more than two treatments at the same time, so that a ranking of competing treatments is not available. Finally, conventional meta-analyses cannot assess subgroup effects reliably [13] although such information is very valuable for clinicians. Theoretically, a single very large trial would overcome these three limitations of conventional meta-analysis. However, the sample size would need to be a multiple of that of the recently published TORCH trial [14] (more than 6000 patients) if subgroup effects were to be investigated.

Since such a trial is very unlikely to become available, a network meta-analysis or individual patient data meta-analysis can be very informative [15]. Such pooled analyses unify evidence from all randomized trials while fully preserving randomization [16-18]. Therefore, our aim was to assess the relative effectiveness of competing inhaled drug regimens for the prevention of exacerbations in patients with stable COPD in a pooled analysis of randomized comparisons. In addition, we assessed whether the effectiveness depend on the severity of COPD, treatment duration, or the definition of an exacerbation (event based or symptom based).

## Methods

### Data sources and selection

We searched the Cochrane Database of Systematic Reviews (Oxford, United Kingdom, 2007, Issue 2), the Database of Abstracts of Reviews of Effects (last search on November 20^th ^2007) and the Health Technology Assessment database (last search November on 20^th ^2007) for randomized trials included in existing systematic reviews (see Additional file 1 for systematic reviews used to identify relevant studies). We based our searches on existing systematic reviews in order to avoid unnecessary duplication of previous work. The existing systematic reviews used extensive search strategies that included several databases such as Medline, EMBASE, CINAHL and LILACS as well as websites of regulatory bodies. In addition, drug companies were approached for unpublished trials. We complemented these searches by entering all included studies into the 'related articles' function of PubMed (last search for trials on November 20^th^, 2007).

We included randomized trials without any language restrictions that were ≥ 4 weeks in duration, included patients with stable COPD, and assessed the effects of long-acting beta-agonists, long-acting anticholinergics, inhaled corticosteroids, or combination treatment (long-acting beta-agonists plus inhaled corticosteroids) on exacerbations with or without placebo control. We included all trials where the proportion of patients with at least one exacerbation during follow-up was reported. We did not include trials where only the mean number of exacerbations per patient year was reported because meta-analysis of such data is prone to bias without individual patient data [19].

**Figure 1:**
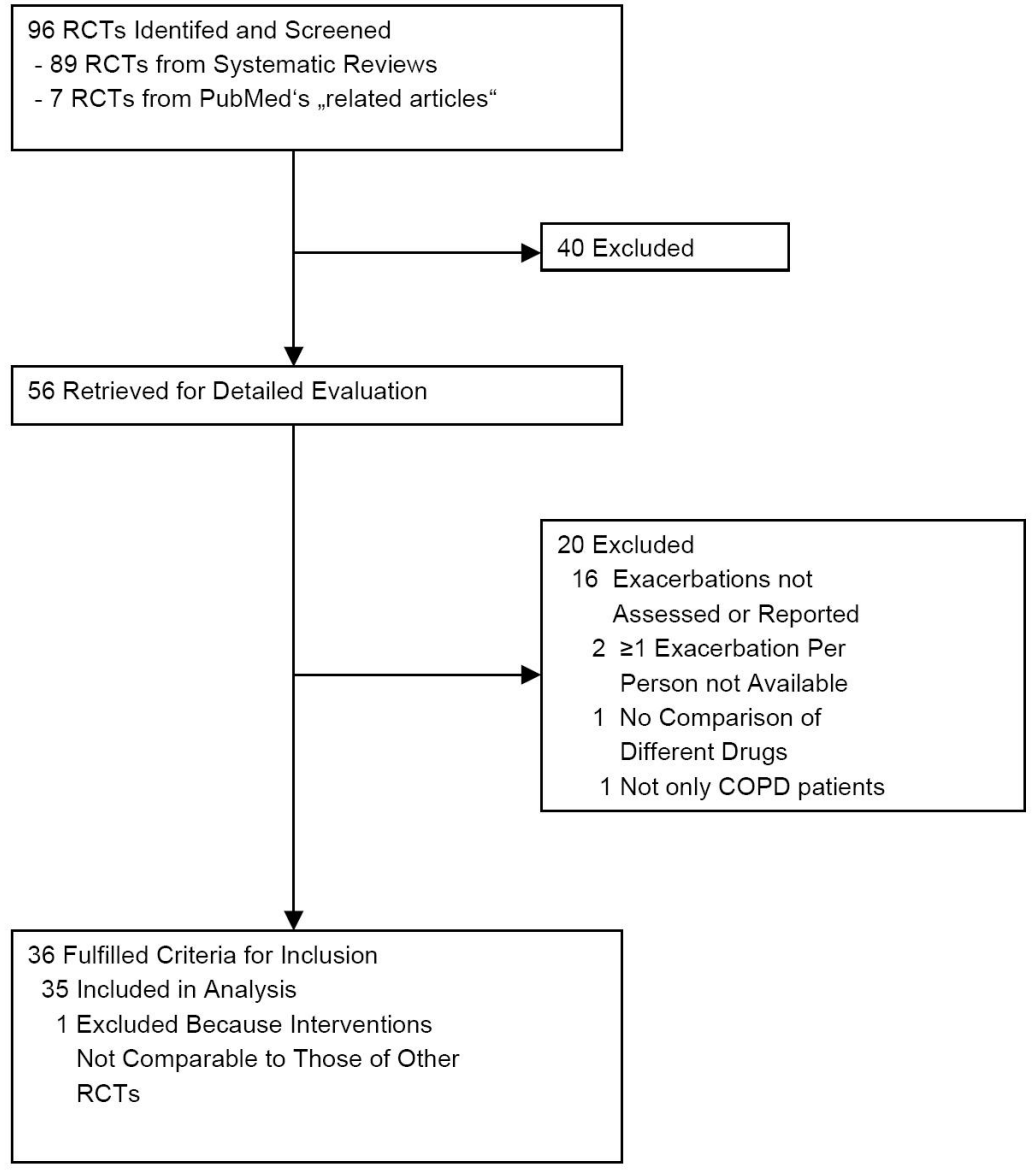
**Study flow from database searches to inclusion oftrials**. 40 trials were excluded after title and abstract screening because they obviously did not fulfill the inclusion criteria (not patients with COPD, patients with unstable COPD, short-acting bronchodilators, treatment duration < 4 weeks, no exacerbations ascertained). Reasons for exclusion for the 20 studies excluded after full text assessment are listed in Additional file 2.

We retrieved all full reports of potentially eligible trials. Two reviewers (research fellows with medical doctor degrees and one and three years of research experience, respectively) independently assessed them and determined their inclusion or exclusion. If the two reviewers disagreed even after discussion, a third reviewer (epidemiologist with MD and PhD degree) arbitrated. Out of 56 trials identified initially, we excluded 20 (Figure 1, see also Additional file 2 for excluded studies). Another trial met the inclusion criteria but it could not be included in the analysis because the treatments (combination of long-acting beta-agonist, long-acting anticholinergic and inhaled corticosteroid versus one or two components), were dissimilar to those in the included trials [20]. We included the remaining 35 randomized trials in the analysis [14,21-54]. One trial [35] was republished together with another trial [24]. We considered the data from both trials as published in the second report [24].

### Data extraction

We focused our analysis on exacerbations. For each trial arm (2 to 4, depending on the trial), one reviewer extracted the number of patients with at least one exacerbation during follow-up and the number of patients with no exacerbation during follow-up (2 × 2, 3 × 2, or 4 × 2 tables). A second and, in case of disagreement, third reviewer, checked the data extraction for correctness. We also recorded whether the studies' definition of exacerbation was event based (physician or emergency room visit, hospital admission) or symptom based (increase of dyspnea, sputum, or cough; for example according to Anthonisen et al [55]) and recorded the severity of exacerbation according to the Operational Classification of Severity of the European Respiratory and American Thoracic Societies (severe or mild to moderate exacerbation defined as requiring inpatient treatment or outpatient treatment, respectively) [9]. We recorded the drugs that were evaluated and classified them into the categories of placebo, long-acting beta-agonists (salmeterol, formoterol), long-acting anticholinergics (tiotropium bromide), and inhaled corticosteroids (budesonide, fluticasone, flunisolide, or beclomethasone), or combined treatment with a long-acting beta-agonist and an inhaled corticosteroid. Finally, we recorded treatment duration in weeks, the patient group's mean age, and mean FEV_1_.

### Study reporting quality assessment

For each trial, two reviewers independently evaluated the quality of reporting for important components of internal validity (Additional file 3). We assessed the method of randomization, concealment of random allocation and whether inclusion criteria were specified in order to judge whether confounding was controlled for by randomization and/or restriction. We recorded blinding of treatment providers and patients to judge the presence of information bias and we recorded whether an intention-to-treat analysis was reported to assess if randomization was maintained throughout the analyses. We resolved discordant scores based on real differences in interpretation through consensus or third party arbitration. We used the quality assessment to judge the validity of the trial results.

### Data synthesis and analysis

Based on 2 × 2 tables from each study (or 3 × 2 and 4 × 2 tables, respectively), we created as many data entries with respective coding for treatment and exacerbation (yes/no) as there were patients in the respective cell. For example, in the study of Brusasco et al [24] there were 156 patients in the placebo group with an exacerbation and 244 patients without an exacerbation, resulting in 156 (exacerbation = yes) and 244 (exacerbation = no) data entries for the 400 patients with placebo. For each of these entries we entered the mean age and FEV_1 _as covariates. For a detailed description of creating such a data set, see Additional file 4.

We performed a logistic regression arm-level analysis with the presence of exacerbation as dependent and the different treatment options as independent variables. We started with placebo treatment as the reference group followed by identical analyses where long-acting beta-agonists, long-acting anticholinergics and inhaled corticosteroids, respectively, served as reference group to which the other treatments were compared. To preserve randomization within each trial, we included a dummy variable for each of the studies. This dummy variable also adjusted for differences in risk profiles and study setup between trials. Second, we conducted stratified analyses to assess whether treatment duration or follow-up, respectively, disease severity (expressed by FEV_1_), or the definition of exacerbation influenced the (relative) treatment effects. We performed separate analyses for trials with a treatment duration of ≤ and > 6 months and≤and > 12 months, for trials with event- or symptom-based exacerbations, and for trials including patients with a mean post-bronchodilator FEV_1 _of ≤ and > 40% and 50% predicted (where available). We did not select 35% predicted as cut-off because there was only one trial [47] with such a low mean FEV_1_. To assess effect modification formally, we tested for interaction by introducing product terms between predictor variables and treatments into the logistic regression analysis. With a random coefficient model, we investigated the presence of any additional variation of the treatment effects due to differences across trials [56]. All analyses were conducted using STATA (STATA™ for Windows, version 9, Stata Corp; College Station, TX).

### Role of the funding source

This work was supported by the Swiss National Science Foundation and The Helmut Horten Foundation. These funding sources did not have any influence on the planning, conduct and reporting of this study.

## Results

### Study characteristics

Table 1 (parts A-E) shows the characteristics of the 35 trials with 26,786 patients. Median sample size per trial was 545 (interquartile range, 253 to 976). On the patient level, median treatment duration was 25 weeks (interquartile range, 12 to 52), median age was 64 years (interquartile range, 63 to 65), and median FEV_1 _42% predicted (interquartile range, 40 to 46). A total of 26 trials (74%) with 23,245 patients used an event-based definition for exacerbations, and nine trials (26%) with 3541 patients had a symptom-based definition. 7201 patients (27%) suffered from at least one exacerbation. A total of 8312 patients received placebo (32 trials), 6357 received long-acting beta-agonists (21 trials), 4764 received a long-acting anticholinergic (11 trials), 3492 an inhaled corticosteroid (12 trials), and 3861 patients combination treatment (8 trials).

**Table 1:** Study characteristics

**Part A**					
**Study**	**Population**	**Inhaled drug treatment**	**Treatment duration (months)**	**Definition of exacerbation**	**Severity of exacerbation**

Baumgartner et al 2007 [48]	*n *= 433 (58% males) Mean age in years: 63 Mean FEV1: 41% predicted	**Group 1**: Salmeterol 42 μg bid **Group 2**: Formoterol 25 μg bid **Group 3**: Placebo	3	Event-based	Moderate and severe

Beeh et al 2006 [21]	*n *= 1639 (76% males) Mean age in years: 62 Mean FEV1: 45% predicted	**Group 1**: Tiotropium 18 μg qd **Group 2**: Placebo	3	Event-based	Moderate and severe

Bourbeau et al 1998 [22]	*n *= 79 (66% males) Mean age in years: 66 Mean FEV1: 43% predicted	**Group 1**: Budesonide 800 μg bid **Group 2**: Placebo	6	Event-based	Moderate and severe

Boyd et al 1997[23]	*n *= 445 (79% males) Mean age in years: 62 Mean FEV1: ≈65% predicted (only FEV1 in liters reported)	**Group 1**: Salmeterol 50 μg bid **Group 2**: Placebo	3.5	Event-based	Moderate and severe

Briggs et al 2005[49]	*n *= 653 (66% males) Mean age in years: 64 Mean FEV1: 38% predicted	**Group 1**: Tiotropium 18 μg qd **Group 2**: Salmeterol 50 μg bid	3	Symptom-based	Moderate and severe

Brusasco et al 2003 [24,35]	*n *= 1207 (76% males) Mean age in years: 64 Mean FEV1: 38% predicted	**Group 1**: Tiotropium 18 μg qd **Group 2**: Salmeterol 50 μg bid **Group3**: Placebo	6	Symptom-based	Moderate and severe

Burge et al 2000 [25]	n = 742 (75% males) Mean age in years: 64 Mean FEV1: 50% predicted	**Group 1**: Fluticasone 500 μg bid **Group 2**: Placebo	36	Event-based	Moderate and severe

Calverley et al 2003 [26]	*n *= 1465 (76% males) Mean age in years: 63 Mean FEV1: 45% predicted	**Group 1**: Salmeterol 50 μg bid **Group 2**: Fluticasone 500 μg bid **Group 3**: Salmeterol 50 μg bid + Fluticasone 500 μg bid **Group 4**: Placebo	12	Event-based	Moderate and severe

**Part B**					

Calverley et al 2003 [27]	*n *= 1022 (73% males) Mean age in years: 64 Mean FEV1: 36% predicted	**Group 1**: Formoterol 9 μg bid **Group 2**: Budesonide 400 μg bid **Group 3**: Formoterol 9 μg bid + Budesonide 320 μg bid **Group 4**: Placebo	12	Event-based	Moderate and severe

Calverley et al 2003 [28]	*n *= 78 (60% males) Mean age in years: 65 Mean FEV1: 40% predicted	**Group 1**: Tiotropium 18 μg qd **Group 2**: Placebo	6	Event-based	Moderate and severe

Calverley et al 2007 [14]	*n *= 6112 (76% males) Mean age in years: 65 Mean FEV1: 44% predicted	**Group 1**: Salmeterol 50 μg bid **Group 2**: Fluticasone 500 μg bid **Group 3**: Salmeterol 50 μg bid + Fluticasone 500 μg bid **Group 4**: Placebo	36	Event-based	Severe

Campbell et al 2005 [29]	*n *= 432 (67% males) Mean age in years: 60 Mean FEV1: 54% predicted	**Group 1**: Formoterol 9 μg bid **Group 2**: Placebo	6	Event-based	Moderate and severe

Casaburi et al 2002 [50]	*n *= 921 (65% males) Mean age in years: 65 Mean FEV1: 36% predicted	**Group 1**: Tiotropium 18 μg qd **Group 2**: Placebo	12	Symptom-based	Moderate and severe

Celli et al 2003 [30]	*n *= 824 (70% males) Mean age in years: 64 Mean FEV1: 42% predicted	**Group 1**: Salmeterol 50 μg bid **Group 2**: Placebo	3	Event-based	Moderate and severe

Chapman et al 2002 [31]	*n *= 408 (64% males) Mean age: not reported Mean FEV1: 45% predicted	**Group 1**: Salmeterol 50 μg bid **Group 2**: Placebo	5.5	Event-based	Moderate and severe

Covelli et al 2004 [32]	*n *= 196 (66% males) Mean age in years: 65 Mean FEV1: 40% predicted	**Group 1**: Tiotropium 18 μg qd **Group 2**: Placebo	3	Symptom-based	Moderate and severe

**Part C**					

Dahl et al 2001 [33]	*n *= 392 (75% males) Mean age in years: 64 Mean FEV1: 45% predicted	**Group 1**: Formoterol 24 μg bid **Group 2**: Placebo	3	Event-based	Severe

Dusser et al 2006 [34]	*n *= 1010 (88% males) Mean age in years: 65 Mean FEV1: 48% predicted	**Group 1**: Tiotropium 18 μg qd **Group 2**: Placebo	12	Event-based	Moderate and severe

Hanania et al 2003 [51]	*n *= 540 (63% males) Mean age in years: 64 Mean FEV1: 42% predicted	**Group 1**: Salmeterol 50 μg bid **Group 2**: Fluticasone 250 μg bid **Group 3**: Salmeterol 50 μg bid + Fluticasone 250 μg bid **Group 4**: Placebo	5.5	Event-based	Moderate and severe

Kardos et al 2007 [52]	*n *= 994 (76% males) Mean age in years: 64 Mean FEV1: 40% predicted	**Group 1**: Salmeterol 50 μg bid **Group 2**: Salmeterol 50 μg bid + Fluticasone 500 μg bid	10	Event-based	Moderate and severe

Littner et al 2000 [36]	*n *= 68 (65% males) Mean age in years: 66 Mean FEV1: 42% predicted	**Group 1**: Tiotropium 18 μg qd **Group 2**: Placebo	1	Symptom-based	Moderate and severe

Mahler et al 1999 [37]	*n *= 278 (74% males) Mean age in years: 63 Mean FEV1: 40% predicted	**Group 1**: Salmeterol 42 μg bid **Group 2**: Placebo	3	Symptom-based	Moderate and severe

Mahler et al 2002 [38]	*n *= 671 (66% males) Mean age in years: 64 Mean FEV1: 41% predicted	**Group 1**: Salmeterol 50 μg bid **Group 2**: Fluticasone 500 μg bid **Group 3**: Salmeterol 50 μg bid + Fluticasone 500 μg bid **Group 4**: Placebo	5.5	Event-based	Moderate and severe

**Part D**					

Niewoehner et al 2005 [39]	*n *= 1829 (99% males) Mean age in years: 68 Mean FEV1: 36% predicted	**Group 1**: Tiotropium 18 μg qd **Group 2**: Placebo	6	Event-based	Moderate and severe

Paggiaro et al 1998 [40]	*n *= 281 (74% males) Mean age in years: 63 Mean FEV1: 57% predicted	**Group 1**: Fluticasone 500 μg bid **Group 2**: Placebo	12	Event-based	Moderate and severe

Paggiaro et al 2006 [41]	*n *= 114 (73% males) Mean age in years: 66 Mean FEV1: 53% predicted	**Group 1**: Flunisolide 1 mg bid **Group 2**: Placebo	6	Event-based	Moderate and severe

Rossi 2002[42]	*n *= 434 (84% males) Mean age in years: 63 Mean FEV1: 47% predicted	**Group 1**: Formoterol 25 μg bid **Group 2**: Placebo	3	Event-based	Moderate and severe

Stockley et al 2006 [43]	*n *= 634 (76% males) Mean age in years: 62 Mean FEV1: 46% predicted	**Group 1**: Salmeterol 50 μg bid **Group 2**: Placebo	12	Event-based	Moderate and severe

Szafranski et al 2000 [44]	*n *= 812 (79% males) Mean age in years: 64 Mean FEV1: 36% predicted	**Group 1**: Formoterol 4.5 μg bid **Group 2**: Budesonide 200 μg bid **Group 3**: Formoterol 4.5 μg bid + Budesonide 160 μg bid **Group 4**: Placebo	12	Event-based	Moderate and severe

Van der Valk et al 2002 [45]	*n *= 244 (84% males) Mean age in years: 64 Mean FEV1: 57% predicted	**Group 1**: Fluticasone 500 μg bid **Group 2**: Placebo	5.5	Event-based	Moderate and severe

**Part E**					

Van Noord et al 2000 [46]	*n *= 92 (88% males) Mean age in years: 65 Mean FEV1: 41% predicted	**Group 1**: Salmeterol 50 μg bid **Group 2**: Placebo	3	Event-based	Moderate and severe

Wadbo et al 2002 [47]	*n *= 121 (53% males) Mean age in years: 64 Mean FEV1: 33% predicted	**Group 1**: Formoterol 18 μg bid **Group 2**: Placebo	3	Symptom-based	Moderate and severe

Wedzicha et al 2008 [53]	*n *= 1323 (83% males) Mean age in years: 64 Mean FEV1: 39% predicted	**Group 1**: Tiotropium 18 μg qd **Group 2**: Salmeterol 50 μg bid + Fluticasone 500 μg bid	24	Event-based	Moderate and severe

Weir et al 1999 [54]	*n *= 98 (72% males) Mean age in years: 66 Mean FEV1: not reported	**Group 1**: Beclomethasone 750–1000 μg bid **Group 2**: Placebo	5.5	Symptom-based	Moderate and severe

### Quality of reporting

The median number of adequately reported aspects of study quality was 4 out of 6 (interquartile range 3–5, Additional file 1). This was largely influenced by the frequent reporting of inclusion criteria and blinding (94.1% of trials). However, the method of randomization (35.3%), concealment of random allocation (29.4%) and an intention to treat analysis (50.0%) were reported less frequently.

### Comparisons of inhaled drug regimes

**Figure 2:**
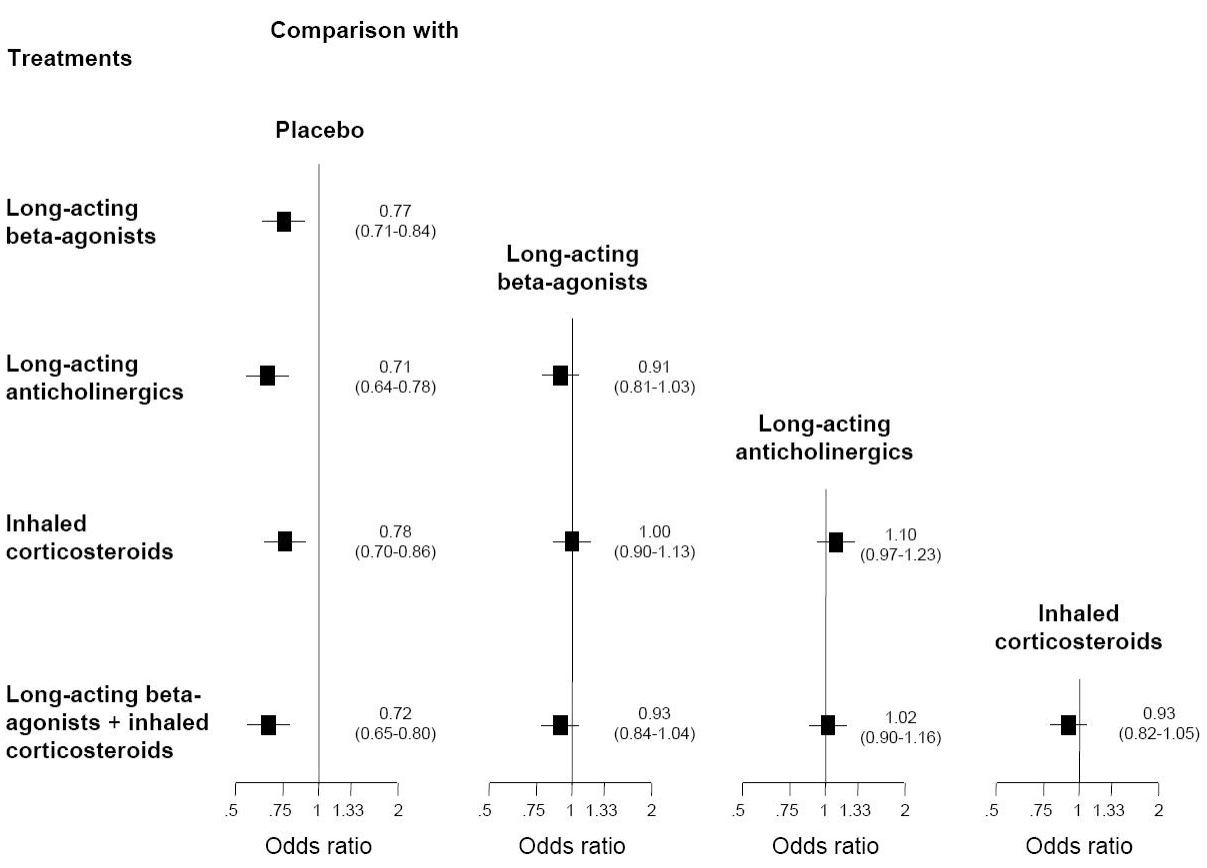
**All comparisons among inhaled drug regimens**. The forest plots show odds ratios (95% confidence intervals) indicating the odds of at least one exacerbation in patients with a drug treatment from the row as compared with treatment from the corresponding column. For example, the odds ratio of 0.91 (0.81 to 1.03) indicates that long-acting anticholinergics are more effective than long-acting beta-agonists, although not significantly so.

All treatments significantly reduced exacerbations when compared with placebo, with odds ratios ranging from 0.71 (95% confidence interval [CI] 0.64 to 0.80) for long-acting anticholinergics to 0.78 (95% CI 0.70 to 0.86) for inhaled corticosteroids (Figure 2). Comparing active drugs among each other, we found no significant differences between long-acting beta-agonists, long-acting anticholinergics, inhaled corticosteroids, and combination treatment. In particular, there were no significant differences between long-acting beta-agonists and long-acting anticholinergics (odds ratio, 0.91; 95% CI 0.81 to 1.03), or between combination treatment and long-acting beta-agonists (odds ratio, 0.93; 95% CI 0.84 to 1.04) or long-acting anticholinergics alone (odds ratio 1.02; 95% CI 0.90 to 1.16).

**Figure 3:**
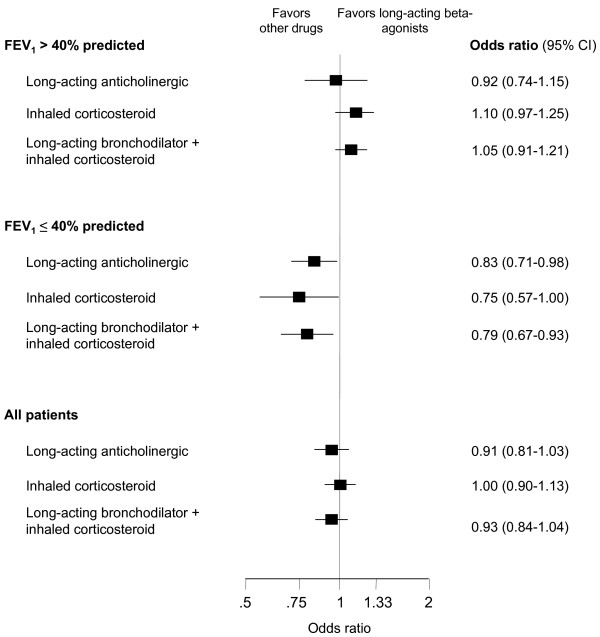
**Treatment comparisons**. Comparisons of long-acting anticholinergics, inhaled corticosteroids and the combination of long-acting beta-agonists + inhaled corticosteroids with long-acting beta-agonists alone stratified for trials including patients with an FEV_1 _> or ≤ 40% predicted.

When we analyzed patients with FEV_1 _≤ 40% (*n *= 9,434, mean FEV_1 _= 37% predicted), long-acting anticholinergics (odds ratio 0.83; 95% CI 0.71 to 0.98), inhaled corticosteroids (odds ratio 0.75; 95% CI 0.57 to 1.00), and combination treatment (odds ratio 0.79; 95% CI 0.67 to 0.93) reduced exacerbations significantly compared with long-acting beta-agonists alone (Figure 3). In patients with FEV_1 _> 40% predicted (*n *= 17,352, mean FEV_1 _= 46% predicted), there were no differences between treatments. Thus, the difference between point estimates in patients with FEV_1 _≤ or > 40% was small for long-acting anticholinergics (0.09) but larger for inhaled corticosteroids (0.35) and combination treatment (0.26). This effect modification was confirmed when we tested for effect modification formally (*P *= 0.46 for long-acting anticholinergics, *P *= 0.02 for inhaled corticosteroids, and *P *= 0.01 for combination treatment). Effects of long-acting anticholinergics and combination treatment did not differ significantly (odds ratio 0.94; 95% CI 0.80 to 1.11). There was no effect modification when stratified for FEV_1 _≤ or > 50%. In the other stratified analyses (stratified for treatment duration of ≤ and > 6 months and ≤ and > 12 months as well as for definition of exacerbation), we did not observe any influence of treatment duration or the definition of exacerbations on (relative) treatment effects.

The random coefficient model showed very similar point estimates and 95% CIs. Compared with the simpler logistic regression model, there was very small extra variability arising from differences of treatment effects across trials (point estimates of interstudy variances range from 10^-6 ^to 10^-9^).

## Discussion and conclusion

Based on 35 trials, our analysis showed that all inhaled drug regimens significantly reduced exacerbations but there were no significant differences between them. Thus, it appears that inhaled corticosteroids provide no additional value in reducing exacerbations when used concurrently with long-acting beta-agonists. However, combination treatment appeared to be more effective than beta-agonists alone in patients with low FEV_1_.

According to the recently published BOLD study, the prevalence of COPD with GOLD stage II to IV in populations over 40 years of age is around 10%, and approximately 80% of these patients have an FEV_1 _≥ 50% predicted (GOLD stage II) [57]. For these patients, our study suggests that single treatment with long-acting beta-agonists or long-acting anticholinergics is not only the treatment of choice for symptom control but also for preventing exacerbations. Although common practice is to prescribe inhaled corticosteroids also [58,59], our findings suggest that these patients do not need the additional medical therapy to reduce exacerbations.

In contrast, in patients with low FEV_1 _(below 40% predicted), long-acting anticholinergics appear to be the most attractive choice. They are as effective as combination treatment in reducing exacerbations but offer advantages in terms of costs and adverse effects [53]. The only head-to-head comparison of a long-acting anticholinergic and combination treatment, the recently published INSPIRE study, identified outcomes in agreement with these findings [53]. The INSPIRE study also showed no clinically relevant differences in health-related quality of life (< 4 points on St. Georges Respiratory Questionnaire). A significant reduction in 2-year mortality was observed with combination treatment (3%) compared with the long-acting anticholinergic (6%). However, this mortality analysis should be interpreted with caution because a closer look at how death was ascertained in the INSPIRE trials reveals that death was only recorded for patients who did not withdraw from treatment (65.5% with combined treatment and 58.3% with long-acting anticholinergic). Thus, it appears that one third of patients did not enter the mortality analysis. Unfortunately, little evidence is available about the combination of long-acting anticholinergics and inhaled corticosteroids; however, a recent Canadian trial indicated that this combination could be the most effective treatment for reducing exacerbations in patients with low FEV_1 _[20].

Exacerbation is not the only outcome that should inform the decision for or against adding inhaled corticosteroids to long-acting bronchodilators. Other outcomes should be considered as well. A recent systematic review found no risk reduction in terms of mortality if an inhaled steroid was added to a long-acting bronchodilator [11]. Health-related quality of life was statistically significantly better after combined treatment (difference of -1.64 units on St. Georges Respiratory Questionnaire; 95% CI -2.28 to -1) but the effects were well under the threshold representing a minimal important difference (4 units) [11]. Arguments for treating COPD solely with long-acting beta-agonists are the substantially lower costs and lower risk for adverse effects such as pneumonia, oral candidiasis, or loss of bone density compared with combination treatment [11,14,53,60]. Integrating and presenting this complex information about benefits and downsides of inhaled drug treatments is challenging. One approach is to use decision aids that are particularly valuable for value-sensitive decisions where the balance of benefits and downsides is not straightforward. As the decision is, in the case of inhaled drug treatment for COPD, only between two treatment options (bronchodilator(s) versus combined treatment), information about the comparisons with placebo could now be excluded for simplicity, since offering no treatment is not in the best interest of the patient. However, whether patients benefit from such informed decision-making requires testing in additional trials.

Unlike previous meta-analyses based on comparisons of inhaled drugs with placebo or, in some instances, with another inhaled drug [4-6,11,12], we argue that the comparative effectiveness of available treatments are of greater interest to physicians [7,8]. To provide estimates for this comparative effectiveness, we used a new analytical approach to pool evidence from all available randomized trials. Such analyses may be, in many instances, the only way to estimate comparative treatment effectiveness; head-to-head comparisons are unavailable, and the sample sizes required to detect small differences are unfeasibly large. For comparison of inhaled COPD drugs, for example, thousands of patients are required just for the main comparison, not even accounting for subgroup effects.

There are other approaches for indirect comparisons [16-18]. An early and important contribution emphasized the importance of preserving randomization [16]. However, that proposed approach only allows for a single head-to-head comparison and not for simultaneous comparison of all treatments under consideration. Two other approaches [17,18] are available, but the complexity of their statistical methods may represent a barrier for their application and interpretation [61]. Our approach is particularly attractive because it is transparent and easily reproducible. The multivariable logistic regression analysis allows for an analysis like that used in any randomized trial while keeping intact the randomization of each trial. The comparisons with placebo showed the same results as those of the meta-analyses of the Clinical Practice Guideline of the American College of Physicians [6]. When the odds ratios are transformed to relative risks [62], the effect estimates are identical to those of a conventional meta-analysis.

A limitation of our study is that we focused on exacerbations and did not consider additional outcomes such as health-related quality of life or mortality. When we planned the current analyses the body of evidence on mortality was too small to provide precise effect estimates, even if pooled. The TORCH trial [14] published in 2007 was the first trial that was powered to assess the effects of inhaled drugs on mortality, but even this very large trial turned out to be too small. Therefore, we decided to postpone analyses on mortality to a point in time where more data would become available. Another limitation is that we performed only a few stratified analyses. For physicians, knowing more about profile-specific effect estimates would be very useful. For example, more detailed information about risk factors for exacerbations, such as previous exacerbations, would enhance understanding about who benefits from the addition of inhaled corticosteroids. Single trials are unlikely to provide such information because of the very large sample sizes they would require for sufficient power to detect effect modification. Individual patient data meta-analysis and pooled analyses may solve this problem to some extent. However, reporting of such patient characteristics is highly variable, and retrieval of individual patient data from primary studies is challenging [63].

A limitation of any approach to pool data on exacerbations is that (relative) treatment effects can only be estimated adequately based on the proportion of patients with at least one exacerbation. Analysis of exacerbation rates expressed as mean exacerbation rates per person-year would offer a more comprehensive use of the data and would be less dependent on the length of follow-up. However, such data cannot be pooled adequately without having access to adequate and fully reported analyses that took within- and between-patient variability in exacerbations into account. But as Suissa points out, most trials are not analyzed and reported adequately[19]. Ideally, an individual patient data meta-analysis would be conducted but it is very challenging to convince investigators of all relevant trials to share their data [63]. The advantage of using the proportion of patients with at least one exacerbation is the limited influence of few patients with many exacerbations on the results. Also, physicians may be more familiar with this format and respective estimates for treatment effects such as odds ratios or relative risks than with mean differences in exacerbation rates. However, there is little evidence on how effect estimates should be presented in order to facilitate the transfer from research into practice.

A common problem of systematic reviews in COPD is poor reporting of clinical and spirometric characteristics of patients enrolled in included trials. It is sometimes difficult to judge whether all patients had COPD, or if some patients had other lung disease such as asthma. Also, there is often no separate reporting for moderate and severe exacerbations, although it would be informative to estimate the effects of inhaled steroids stratified for the severity of exacerbations. COPD is a heterogeneous disease and it is important that future studies provide more information about their patients, including clinical characteristics, lung function data and also information on co-treatments.

The results of our study may support physicians in selecting inhaled drug treatments for patients with COPD. In general, long-acting beta-agonists or anticholinergics appear to be the treatment of choice to reduce exacerbation rates. Adding an inhaled corticosteroid does not provide additional protection from exacerbations. In patients with low FEV_1_, combination treatment and long-acting anticholinergics should be favored because they reduce the risk for exacerbations more than single treatment with a long-acting beta-agonist. Our analyses, together with the existing meta-analyses that considered additional outcomes, inform patients and physicians to balance the benefits and downsides of different inhaled drug treatments for COPD.

## Competing interests

None of the authors has any competing interests related to the content of this paper. Dr. Kleijnen received restricted research grants from Pfizer Ltd.

## Authors' contributions

All authors had full access to all of the data in the study. MAP takes responsibility for the integrity of the data and accuracy of the data analysis. MAP, LMB, JK, and AGK undertook Study concept and design. MAP, LMB and AGK were responsible for data acquisition. MAP, LMB, JK, GtR and AGK performed Analysis and interpretation of data. MAP, LMB and AGK drafted the manuscript. JK and GtR undertook critical revision of the manuscript for important intellectual content. MAP, LMB, GtR and AGK performed the statistical analysis. JK and AGK supervised the study. All authors have read and approved the final manuscript.

## Pre-publication history

The pre-publication history for this paper can be accessed here:



## Supplementary Material

Additional file 1 **Appendix 1.** Existing systematic reviews used for identification of articles. Click here for file

Additional file 2 **Appendix 2.** Excluded studies. Click here for file

Additional file 3 **Appendix 3.** Quality assessment. Click here for file

Additional file 4 **Appendix 4.** Approach to create new data set with n data entries where n is the total number of included patients. Click here for file
