# Supplementary material for: Inhaled drugs to reduce exacerbations in patients with chronic obstructive pulmonary disease: a network meta-analysis
Source: BMC Med. 2009 Jan 14;7:2. doi: 10.1186/1741-7015-7-2 (PMC2636836; doi:10.1186/1741-7015-7-2)
Supplement: Additional file 1 — Appendix 1. Existing systematic reviews used for identification of articles. [file 1741-7015-7-2-S1.doc]

**Appendix 1: Existing systematic reviews used for identification of articles**

1. Nannini L, Cates C, Lasserson T, Poole P: **Combined corticosteroid and long-acting beta-agonist in one inhaler versus placebo for chronic obstructive pulmonary disease**. *Cochrane database of systematic reviews (Online)* 2007(4):CD003794.
2. Yang IA, Fong KM, Sim EH, Black PN, Lasserson TJ: **Inhaled corticosteroids for stable chronic obstructive pulmonary disease**. *Cochrane database of systematic reviews (Online)* 2007(2):CD002991.
3. Wilt TJ, Niewoehner D, Macdonald R, Kane RL: **Management of Stable Chronic Obstructive Pulmonary Disease: A Systematic Review for a Clinical Practice Guideline**. *Ann Intern Med* 2007, **147**(9):639-653.
4. Nannini L, Cates C, Lasserson T, Poole P: **Combined corticosteroid and long-acting beta-agonist in one inhaler versus long-acting beta-agonists for chronic obstructive pulmonary disease**. *Cochrane database of systematic reviews (Online)* 2007(4):CD006829.
5. Nannini L, Cates C, Lasserson T, Poole P: **Combined corticosteroid and long-acting beta-agonist in one inhaler versus inhaled steroids for chronic obstructive pulmonary disease**. *Cochrane database of systematic reviews (Online)* 2007(4):CD006826.
6. Appleton S, Poole P, Smith B, Veale A, Lasserson TJ, Chan MM. **Long-acting beta2-agonists for poorly reversible chronic obstructive pulmonary disease.** *Cochrane database of systematic reviews (Online)* 2006(3): CD 001104.
