# Supplementary material for: Inhaled drugs to reduce exacerbations in patients with chronic obstructive pulmonary disease: a network meta-analysis
Source: BMC Med. 2009 Jan 14;7:2. doi: 10.1186/1741-7015-7-2 (PMC2636836; doi:10.1186/1741-7015-7-2)
Supplement: Additional file 2 — Appendix 2. Excluded studies. [file 1741-7015-7-2-S2.doc]

Appendix 2: Excluded studies

| **Study** | **Reason for exclusion** |
| --- | --- |
| 1. Aalbers R, Ayres J, Backer V, et al. Formoterol in patients with chronic obstructive pulmonary disease: a randomized, controlled, 3-month trial. European Respiratory Journal 2002;19(5):936-43. | Exacerbations not assessed. |
| 2. Auffarth B, Postma DS, De Monchy JGR, Van der Mark TW, Boorsma M, Koeter GH. Effects of inhaled budesonide on spirometric values, reversibility, airway responsiveness, and cough threshold in smokers with chronic obstructive lung disease. Thorax 1991(5):372-7. | Exacerbations not assessed. |
| 3. Casaburi R, Briggs DD, Jr., Donohue JF, Serby CW, Menjoge SS, Witek TJ, Jr. The spirometric efficacy of once-daily dosing with tiotropium in stable COPD: a 13-week multicenter trial. The US Tiotropium Study Group. Chest 2000;118(5):1294-302. | Exacerbations not assessed. |
| 4. Celli B, ZuWallack R, Wang S, Kesten S. Improvement in resting inspiratory capacity and hyperinflation with tiotropium in COPD patients with increased static lung volumes. Chest;2003 Nov; 124(5):1743-8. | Exacerbations not assessed. |
| 5. Fukuchi Y, Nagai A, Seyama K, et al. Clinical efficacy and safety of transdermal tulobuterol in the treatment of stable COPD: An open-label comparison with inhaled salmeterol. Treatments in Respiratory Medicine 2005(6):447-55. | Exacerbations not assessed. |
| 6. Gupta RK, Chhabra SK. An evaluation of salmeterol in the treatment of chronic obstructive pulmonary diseases. Indian Journal of Chest Diseases & Allied Sciences 2002;44(3):165-72. | Exacerbations not assessed. |
| 7. Jones PW, Bosh TK. Quality of life changes in COPD patients treated with salmeterol. American Journal of Respiratory & Critical Care Medicine 1997;155(4):1283-9. | Exacerbations not assessed. |
| 8. McNicholas WT, Calverley PM, Lee A, Edwards JC, Tiotropium Sleep Study in CI. Long-acting inhaled anticholinergic therapy improves sleeping oxygen saturation in COPD. European Respiratory Journal 2004;23(6):825-31. | No comparison of different drugs. |
| 9. Mirici A, Bektas Y, Ozbakis G, Erman Z. Effect of inhaled corticosteroids on respiratory function tests and airway inflammation in stable chronic obstructive pulmonary disease: A randomised, double-blind, placebo-controlled clinical trial. Clinical Drug Investigation 2001(12):835-42. | Exacerbations not assessed. |
| 10. O'Donnell DE, Fluge T, Gerken F, et al. Effects of tiotropium on lung hyperinflation, dyspnoea and exercise tolerance in COPD. European Respiratory Journal 2004;23(6):832-40. | Exacerbations not assessed. |
| 11. Ozol D, Aysan T, Solak ZA, Mogulkoc N, Veral A, Sebik F. The effect of inhaled corticosteroids on bronchoalveolar lavage cells and IL-8 levels in stable COPD patients. Respiratory Medicine 2005;99(12):1494-500. | Exacerbations not assessed. |
| 12. Pauwels RA, Lofdahl CG, Laitinen LA, et al. Long-term treatment with inhaled budesonide in persons with mild chronic obstructive pulmonary disease who continue smoking. European Respiratory Society Study on Chronic Obstructive Pulmonary Disease. New England Journal of Medicine 1999;340(25):1948-53. | Exacerbations not assessed. |
| 13. Renkema TE, Schouten JP, Koeter GH, Postma DS. Effects of long-term treatment with corticosteroids in COPD. Chest 1996;109(5):1156-62. | ≥1 exacerbation per person not available. |
| 14. Rutgers SR, Koeter GH, van der Mark TW, Postma DS. Short-term treatment with budesonide does not improve hyperresponsiveness to adenosine 5'-monophosphate in COPD. American Journal of Respiratory & Critical Care Medicine 1998; 157: 880-886. | Exacerbations not assessed. |
| 15. Thompson AB, Mueller MB, Heires AJ, et al. Aerosolized beclomethasone in chronic bronchitis. Improved pulmonary function and diminished airway inflammation. American Review of Respiratory Disease 1992;146(2):389-95. | Exacerbations not assessed. |
| 16. Verhoeven GT, Hegmans JPJJ, Mulder PGH, Bogaard JM, Hoogsteden HC, Prins JB. Effects of fluticasone propionate in COPD patients with bronchial hyperresponsiveness. Thorax 2002(8):694-700. | Exacerbations not assessed. |
| 17. Vestbo J, Sorensen T, Lange P, Brix A, Torre P, Viskum K. Long-term effect of inhaled budesonide in mild and moderate chronic obstructive pulmonary disease: a randomised controlled trial. Lancet 1999;353(9167):1819-23. | ≥1 exacerbation per person not available. |
| 18. Yildiz F, Basyigit I, Yildirim E, Boyaci H, Ilgazli A. Does addition of inhaled steroid to combined bronchodilator therapy affect health status in patients with COPD? Respirology 2004;9(3):352-5. | Exacerbations not assessed. |
| 19. The Lung Health Study: Effect of inhaled triamcinolone on the decline in pulmonary function in chronic obstructive pulmonary disease. The New England journal of medicine 2000;343(26):1902-9. | Exacerbations not assessed. |
| 20. van den Boom G, Rutten-van Molken MP, Molema J, Tirimanna PR, van Weel C, van Schayck CP: The cost effectiveness of early treatment with fluticasone propionate 250 microg twice a day in subjects with obstructive airway disease. Results of the DIMCA program. American journal of respiratory and critical care medicine 2001, 164(11):2057-2066. | Not only COPD |
