# Supplementary material for: Inhaled drugs to reduce exacerbations in patients with chronic obstructive pulmonary disease: a network meta-analysis
Source: BMC Med. 2009 Jan 14;7:2. doi: 10.1186/1741-7015-7-2 (PMC2636836; doi:10.1186/1741-7015-7-2)
Supplement: Additional file 3 — Appendix 3. Quality assessment. [file 1741-7015-7-2-S3.doc]

Appendix 3: Quality assessment

| **Description of**  **Study** | **Method of randomisation** | **Concealment of random allocation** | **Inclusion criteria specified** | **Blinding of patient** | **Blinding of treatment provider** | **Intention to treat analysis** |
| --- | --- | --- | --- | --- | --- | --- |
| Baumgartner 2007(48) | 0 | 0 | 1 | 1 | 1 | 1 |
| Beeh 2006(21) | 0 | 0 | 1 | 1 | 1 | 1 |
| Bourbeau 1998(22) | 1 | 1 | 1 | 1 | 1 | 0 |
| Boyd 1997(23) | 0 | 0 | 1 | 1 | 1 | 0 |
| Briggs 2005(49) | 0 | 0 | 1 | 1 | 1 | 0 |
| Brusasco 2003(24, 35) | 0 | 0 | 1 | 1 | 1 | 0 |
| Burge 2000(25) | 1 | 1 | 1 | 1 | 1 | 0 |
| Calverley 2003(26) | 1 | 1 | 1 | 1 | 1 | 1 |
| Calverley 2003(27) | 0 | 0 | 1 | 1 | 1 | 1 |
| Calverley 2003(28) | 0 | 0 | 1 | 1 | 1 | 0 |
| Calverley 2007(14) | 1 | 1 | 1 | 1 | 1 | 1 |
| Campbell 2005(29) | 1 | 0 | 1 | 1 | 1 | 1 |
| Casaburi 2002(50) | 0 | 0 | 1 | 1 | 1 | 0 |
| Celli 2003(30) | 1 | 1 | 1 | 1 | 1 | 0 |
| Chapman 2002(31) | 1 | 0 | 1 | 1 | 1 | 1 |
| Covelli 2004(32) | 0 | 0 | 1 | 1 | 1 | 0 |
| Dahl 2001(33) | 0 | 0 | 1 | 1 | 1 | 1 |
| Dusser 2006(34) | 0 | 0 | 1 | 1 | 1 | 1 |
| Hanania 2003(51) | 0 | 0 | 1 | 1 | 1 | 0 |
| Kardos 2007(52) | 0 | 1 | 1 | 1 | 1 | 1 |
| Littner 2000(36) | 0 | 0 | 1 | 1 | 1 | 0 |
| Mahler 1999(37) | 0 | 0 | 1 | 1 | 1 | 0 |
| Mahler 2002(38) | 0 | 0 | 1 | 1 | 1 | 0 |
| Niewoehner 2005(39) | 1 | 1 | 1 | 1 | 1 | 1 |
| Paggiaro 1998(40) | 1 | 1 | 1 | 1 | 1 | 1 |
| Paggiaro 2006(41) | 0 | 0 | 1 | 1 | 1 | 0 |
| Rossi 2002(42) | 0 | 0 | 1 | 1 | 1 | 1 |
| Stockley 2006(43) | 1 | 1 | 1 | 1 | 1 | 1 |
| Szafranski 2000(44) | 0 | 0 | 1 | 1 | 1 | 1 |
| Van der Valk 2002(45) | 1 | 0 | 1 | 1 | 1 | 1 |
| Van Noord 2000(46) | 0 | 0 | 1 | 1 | 1 | 0 |
| Wadbo 2002(47) | 0 | 0 | 0 | 0 | 0 | 0 |
| Wedzicha 2008(53) | 1 | 1 | 1 | 1 | 1 | 1 |
| Weir 1999(54) | 0 | 0 | 0 | 0 | 0 | 0 |

0 = not adequalety described; 1 = adequalety described
