# Supplementary material for: Inhaled drugs to reduce exacerbations in patients with chronic obstructive pulmonary disease: a network meta-analysis
Source: BMC Med. 2009 Jan 14;7:2. doi: 10.1186/1741-7015-7-2 (PMC2636836; doi:10.1186/1741-7015-7-2)
Supplement: Additional file 4 — Appendix 4. Approach to create new data set with n data entries where n is the total number of included patients. [file 1741-7015-7-2-S4.doc]

**Appendix 4: Approach to create new data set with n data entries where n is the total number of included patients**
